# Elevated Serum Growth Differentiation Factor 15 Levels in Hyperthyroid Patients

**DOI:** 10.3389/fendo.2018.00793

**Published:** 2019-01-09

**Authors:** Jiejie Zhao, Min Li, Ying Chen, Shengjie Zhang, Hao Ying, Zhiyi Song, Yan Lu, Xiaoying Li, Xuelian Xiong, Jingjing Jiang

**Affiliations:** ^1^Department of Endocrinology and Metabolism, Fudan Institute of Metabolic Diseases, Zhongshan Hospital, Fudan University, Shanghai, China; ^2^CAS Key Laboratory of Nutrition, Metabolism and Food Safety, Shanghai Institutes for Biological Sciences, University of Chinese Academy of Sciences, Chinese Academy of Sciences, Shanghai, China; ^3^Department of Endocrinology and Metabolism, Shanghai General Hospital, Shanghai Jiao Tong University School of Medicine, Shanghai, China

**Keywords:** growth differentiation factor 15, hyperthyroidism, thyroid hormone, cytokine, brown adipose tissue

## Abstract

**Background:** Recent studies have shown that growth differentiation factor 15 (GDF15), a member of the transforming growth factor-β (TGF-β)/bone morphogenetic protein (BMP) superfamily, plays an important role in appetite, type 2 diabetes, and cardiovascular diseases. Since thyroid hormone has pleiotropic effects on whole-body energy metabolism, we aimed to explore the effect of thyroid hormone on circulating GDF15 levels in humans and GDF15 genes expression in C57BL/6 mice.

**Methods:** A total of 134 hyperthyroid patients and 105 healthy subjects were recruited. Of them, 43 hyperthyroid patients received thionamide treatment for 3 months until euthyroidism. Serum GDF15 levels were determined using the enzyme-linked immunosorbent assay (ELISA) method. To determine the source for the increased circulating GDF15, C57BL/6 mice were treated with T3, and GDF15 gene expressions in the liver, skeletal muscle, brown adipose tissue (BAT), inguinal white adipose tissue (iWAT), epididymal white adipose tissue (eWAT) were analyzed by quantitative real-time polymerase chain reaction (PCR).

**Results:** Serum GDF15 levels were significantly elevated in hyperthyroid patients as compared with healthy subjects (326.06 ± 124.13 vs. 169.24 ± 82.96 pg/mL; *P* < 0.001). After thionamide treatment, GDF15 levels in hyperthyroid patients declined markedly from 293.27 ± 119.49 to 118.10 ± 71.83 pg/mL (*P* < 0.001). After adjustment for potential confounders, serum GDF15 levels were independently associated with hyperthyroidism. T3 treatment increased GDF15 expression in the brown adipose tissue of C57BL/6 mice.

**Conclusions:** Serum GDF15 levels were elevated in patients with hyperthyroidism and declined after thionamide treatment. Thyroid hormone treatment upregulated GDF15 expression in mice. Therefore, our results present the clinical relevance of GDF15 in humans under the condition of hyperthyroidism.

## Introduction

Thyroid hormone plays a crucial role in controlling metabolic rate, adaptive thermogenesis, fatty acid, and cholesterol homeostasis through regulation of target genes in the liver, adipose tissue and skeletal muscle (1, 2). As a result, hyperthyroidism, characterized by excess thyroid hormone, displays increased energy expenditure, accelerated lipolysis and weight loss, despite of increased food intake. Mechanistic studies demonstrate that thyroid hormone could promote metabolic rate and thermogenesis via binding to two thyroid hormone receptors (TRs), namely TRα and TRβ, which consists of four isoforms (α1, β1, β2, and β3) (1, 2). TRα is widely expressed with high expression in the heart, skeletal muscle, and brown adipose tissue (BAT), whereas TRβ is predominately expressed in the brain, liver and kidney. In the presence of thyroid hormone, TRs bind to thyroid hormone response element (TRE) in the regulatory regions of target genes to activate or repress their transcription and expression (1, 2). Activation of TRs by thyroid hormone directly up-regulate the mRNA expression of uncoupling protein 1 (UCP-1) in BAT to promote thermogenesis and carnitine palmitoyltransferase 1α (CPT-1α) in liver to promote fatty acid oxidation (3–5). Besides, recent studies indicate that some cytokines, including fibroblast growth factor 21 (FGF21), Fetuin A and irisin, may mediate the action of thyroid hormone (6–9), suggesting the importance of indirect mechanisms of thyroid hormone signaling in the regulation of energy homeostasis.

Growth differentiation factor 15 (GDF15), a divergent member of the transforming growth factor-β (TGF-β) superfamily, was initially identified as a novel macrophage inhibitory cytokine (10). Subsequent studies found that increased expression and high circulating levels of GDF15 acted as a critical driver of cancer cachexia, largely by decreasing appetite (11, 12). Strong interests emerged when several groups found that the GDNF family receptor α-like (GFRAL) is the receptor for GDF15 to mediate its action in suppressing food intake (13–16). In addition, GDF15 modulates metabolic activity by increasing thermogenesis and energy expenditure, lipolysis and oxidative metabolism, through up-regulation of key thermogenic (UCP-1 and PGC-1α) and lipolytic genes (ATGL and HSL) in BAT and WAT (17). Consistently, adeno-associated virus-mediated overexpression of GDF15 or recombinant GDF15 treatments reduces the adiposity and improves insulin resistance and glucose intolerance in various metabolic disease models (18, 19). On the other hand, GDF15 knockout mice weighed more and had increased adiposity, reduced glucose tolerance, lower locomotor activity and lower metabolic rate than wild-type mice (20, 21). Overall, these studies indicate that GDF15 plays a major role in the regulation of energy substrate utilization or energy expenditure (22).

Due to some similarities in metabolic actions of thyroid hormone and GDF15, especially in preventing obesity and increasing thermogenesis, we therefore tested the hypothesis that thyroid hormone might increase circulating GDF15 levels in humans and further confirmed this regulatory axis in mice.

## Materials and Methods

### Human Subjects

A total of 134 patients with hyperthyroidism and 105 healthy subjects were recruited from the Department of Endocrinology and Metabolism, Zhongshan Hospital, Fudan University (Shanghai, China). Of those, 43 hyperthyroid patients received thionamide treatment for 3 months. Hyperthyroidism were diagnosed according to typical clinical characteristics, including elevated serum TH, reduced TSH, and elevated serum TSH receptor antibody (TRAb) levels. Subjects who had diabetes, cancer, pregnancy, lactation, subacute thyroiditis, abnormal liver function (serum alanine transaminase [ALT], glutamic oxalacetic transaminase [AST] increased by 1.5-fold), abnormal renal function or infectious diseases were excluded. Blood samples were collected after a 12-h of overnight fast, and serum was stored at −80°C for GDF15 and biochemical assays. The study protocol was approved by the Human Research Ethical Committee of Zhongshan Hospital. Written informed consent was obtained from each participant.

### Biochemical Measurements

Serum biochemical measurements were determined on a Hitachi 7600 analyzer (Hitachi, Ltd). Plasma glucose was measured using the glucose oxidase method. Human serum insulin, free T3, free T4, and TSH concentrations were measured using electrochemiluminescence immunoassay (Roche Diagnostics). Human and murine serum GDF15 concentrations were measured using ELISA kits (Raybiotech, USA), according to the manufacturer's instructions. Murine serum total T3 concentrations were measured by ELISA (Calbiotech, USA) following the manufacturer's instructions.

### Animal Experiments

Male C57BL/6 mice aged 10 weeks were purchased from the Shanghai Laboratory Animal Company (SLAC). All mice were housed at 21°C ± 1°C with humidity of 55 ± 10% and a 12-h light/12-h dark cycle. Fifteen Mice were injected intraperitoneally with either vehicle control (saline) or 3,3′,5-Triiodo-L-thyronine (0.5 mg/kg, T2877, Sigma-Aldrich, St. Louis, MO, USA) for 4 hr (single injection) or 5 days (once daily; 5 mice per group). The animal protocol was reviewed and approved by the Animal Care Committee of Zhongshan Hospital.

### RNA Extraction and Quantitative Real-Time PCR

Total RNA was isolated from tissues (liver, skeletal muscle, and adipose tissues) of mice using TRIzol (Invitrogen, Carlsbad, CA, USA) according to the manufacturer's instructions. First-strand complementary DNA (cDNA) synthesis was performed for each RNA sample using the Promega Reverse Transcription System (Madison, WI, USA). Oligo dT was used to prime cDNA synthesis. In order to quantify the transcripts of interest genes, quantitative real-time PCR was performed using a SYBR Green Premix Ex Taq (Takara, Shiga, Japan) on Light Cycler480 (Roche, Switzerland). PCR conditions included an initial holding period at 95°C for 5 min, followed by a two-step PCR program consisting of 95°C for 5 s and 60°C for 30 s for 45 cycles. The mRNA levels of GDF15 were normalized to 36B4 internal control gene. Expression data was analyzed according to the 2^−ΔΔCt^ method. Primers were selected from PrimerBank (https://pga.mgh.harvard.edu/primerbank/) and listed as follows: GDF15 (Forward: 5′-CTGGCAATGCCTGAACAACG-3′; Reverse: 5′-GGTCGGGACTTGGTTCTGAG-3′); Spot14 (Forward: 5′-TGCTAACGAAACGCTATCC-3′; Reverse: 5′-TTCTACACAGTGCTCT TGG-3′); 36B4 (Forward: 5′-AGATTCGGGATATGCTGTTGGC-3′; Reverse: 5′-TCGGGTCCTAGACCAGTGT TC-3′).

### Statistical Analysis

All statistical analyses were performed using SAS 9.3 (SAS Institute, Cary, NC). Variables were presented as mean ± standard deviation (SD). χ^2^ and one-way ANOVA tests were used for comparison of categorical and continuous variables, respectively. The Student's paired *t*-test was used for comparison of categorial variables before and after antithyroid treatment. Pearson's correlation analyses were used to examine the relationship between serum GDF15 levels and other parameters. Multivariable logistic regression was used to calculate the adjusted ORs and 95% CIs. A two-side *P* < 0.05 was considered statistically significant.

## Results

### Serum GDF15 Levels in Patients With Hyperthyroidism

The clinical characteristics of human subjects in this study are displayed in Table 1, which showed that patients with hyperthyroidism exhibited much higher free T3, free T4, increased heart rate, alanine transaminase (ALT) and aspartate transaminase (AST), decreased body mass index (BMI), total cholesterol (TC), and thyroid stimulating hormone (TSH). However, fasting insulin (FINS), fasting plasma glucose (FPG) and triglyceride (TG) levels were comparable between the two groups. Serum GDF15 levels were dramatically increased in hyperthyroid patients compared with healthy subjects (Table 1). In 43 patients, after thionamide treatment, serum free T3, free T4, ALT and AST decreased, whereas body weight, TC, HDL-c, and TSH increased. Accordingly, serum GDF15 concentrations were markedly decreased (Table 2).

**Table 1:** Clinical and biochemical features in healthy and hyperthyroid subjects.

	**Healthy**	**Hyperthyroid**	***P-*value**
	**subjects**	**patients**	
*N*	105	134	
Age (y)	33.23 ± 4.65	34.11 ± 5.38	0.172
Gender (M/F)	68/37	89/45	0.387
BMI (kg/m2)	22.67 ± 1.49	20.92 ± 2.12	<0.001
Heart rate (bpm)	76.20 ± 10.59	109.42 ± 13.23	<0.001
SBP (mmHg)	116.28 ± 14.45	122.05 ± 16.92	0.51
DBP (mmHg)	71.32 ± 10.63	75.89 ± 8.67	0.052
ALT (U/L)	23.73 ± 5.32	34.87 ± 9.71	<0.001
AST (U/L)	26.87 ± 9.87	41.34 ± 11.49	<0.001
TBIL (mmol/L)	13.07 ± 5.79	13.94 ± 507	0.214
DBIL (mmol/L)	2.91 ± 1.04	2.69 ± 1.31	0.159
FINS (mm/L)	10.54 ± 4.11	12.47 ± 4.73	<0.01
FPG (mM)	5.19 ± 0.48	5.30 ± 0.58	0.123
TG (mM)	1.12 ± 0.31	1.17 ± 0.34	0.244
TC (mM)	4.59 ± 0.53	3.48 ± 0.78	<0.001
HDL-c (mM)	1.22 ± 0.34	1.18 ± 0.44	0.386
Free T3 (pM)	5.34 ± 0.42	31.05 ± 12.08	<0.001
Free T4 (pM)	16.44 ± 2.83	82.21 ± 35.88	<0.001
TSH (mIU/L)	1.76 ± 0.94	0.007 ± 0.01	<0.001
GDF15 (pmol/L)	169.24 ± 82.96	326.06 ± 124.13	<0.001

*BMI, body mass index; SBP, systolic blood pressure; DBP, diastolic blood pressure; ALT, alanine transaminase; AST, aspartate transaminase; TBIL, total bilirubin; DBIL, direct bilirubin; FINS, fasting insulin; FPG, fasting plasma glucose; TG, triglyceride; TC, Total cholesterol; HDL, high-density lipoprotein; TSH, thyroid stimulating hormone; GDF15, growth differentiation factor 15. Data are presented as means ± standard deviation (SD)*.

**Table 2:** Serum GDF15 levels before and after thionamide treatment in hyperthyroid subjects.

	**Before**	**After**	***P-*value**
*N*	43	43	
Weight (kg)	52.23 ± 6.26	57.00 ± 7.33	<0.01
ALT (U/L)	37.81 ± 15.92	20.98 ± 9.32	<0.001
AST (U/L)	30.98 ± 12.28	20.63 ± 6.81	<0.001
TBIL (mmol/L)	14.66 ± 7.47	12.08 ± 4.32	0.053
DBIL (mmol/L)	4.88 ± 3.25	3.56 ± 1.61	0.019
FINS (mm/L)	10.69 ± 13.1	8.85 ± 6.81	0.416
FPG (mM)	5.14 ± 0.76	5.01 ± 0.44	0.335
TG (mM)	0.99 ± 0.68	1.11 ± 0.62	0.392
TC (mM)	3.66 ± 0.87	5.50 ± 1.01	<0.001
HDL-c (mM)	1.37 ± 0.39	1.66 ± 0.32	<0.001
Free T3 (pM)	26.62 ± 19.40	4.53 ± 0.83	<0.001
Free T4 (pM)	66.24 ± 32.78	13.05 ± 2.63	<0.001
TSH (mIU/L)	0.011 ± 0.016	0.86 ± 1.28	<0.001
GDF15 (pmol/L)	293.27 ± 119.49	118.10 ± 71.83	<0.001

*Data are presented as means ± standard deviation (SD)*.

### Correlations of Serum GDF15 With Hyperthyroidism and Metabolic Parameters

**Figure 1:**
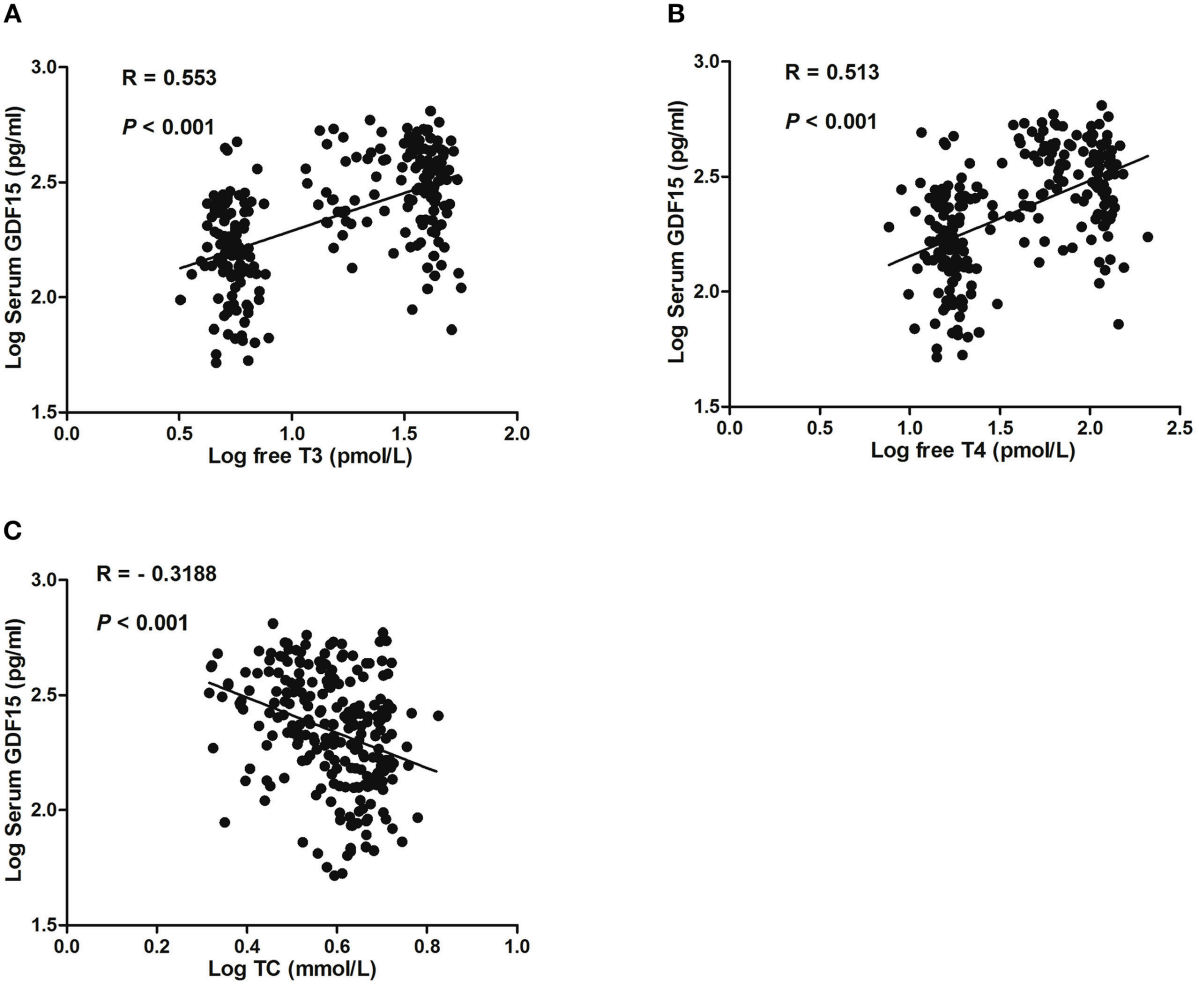
Correlation of GDF15 with serum levels of free T3 **(A)**, free T4 **(B)**, and total cholesterol (TC) **(C)**.

In total hyperthyroid patients and healthy subjects, serum GDF15 levels were positively correlated with serum free T3 and free T4 (Figures 1A,B). Moreover, serum GDF15 levels were negatively correlated with serum TC level (Figure 1C), whereas no significant correlation between serum GDF15 and sex, age, FPG, FINS, and TG was observed (Data not shown).

### Association of Serum GDF15 Levels With Hyperthyroidism

Table 3 shows the unadjusted and adjusted ORs with associated 95% CI of serum GDF15 for hyperthyroidism. Hyperthyroidism were diagnosed according to typical clinical characteristics, including elevated serum TH, reduced TSH, and elevated serum TSH receptor antibody (TRAb) levels. In model 1, with no adjustment for any confounding factor, serum GDF15 levels were significantly associated with hyperthyroidism (OR [95% CI], 6.652 (4.184–11.353); *P* < 0.001). In model 2, after adjustment for age, gender and BMI, serum GDF15 levels were also significantly associated with hyperthyroidism (OR [95% CI], 8.225 (4.805–15.380); *P* < 0.001). In model 3, after further adjustment for ALT, AST, FBG and TC, Serum GDF15 levels remained independently associated with hyperthyroidism, with the adjusted OR (95% CI) of 13.193 (4.754–55.090; *P* < 0.001).

**Table 3:** ORs for association between serum GDF15 levels and hyperthyroidism with the use of three logistic regression models.

**Variable**	**OR (95% CI)**	***P*-value**
**MODEL 1**		
GDF15^a^	6.652 (4.184–11.353)	<0.001
**MODEL 2**		
Age	1.019 (0.946–1.098)	0.622
Gender (female vs. male)	0.517 (0.230–1.161)	0.110
BMI	0.533 (0.424–0.668)	<0.001
GDF15^a^	8.225 (4.805–15.380)	<0.001
**MODEL 3**		
Age	0.903 (0.769– 1.060)	0.214
Gender (female vs. male)	0.249 (0.050–1.236)	0.089
BMI	0.420 (0.256–0.691)	0.0006
ALT	1.299 (1.127–1.498)	0.0003
AST	1.080 (0.995–1.173)	0.065
FPG	6.416 (0.848–48.567)	0.072
TC	0.030 (0.006–0.140)	<0.001
GDF15^a^	13.193 (4.754–55.090)	<0.001

a *OR and 95% CI expressed as per SD increase of GDF15*.

### Thyroid Hormone Promotes GDF15 Expression in Mice

**Figure 2:**
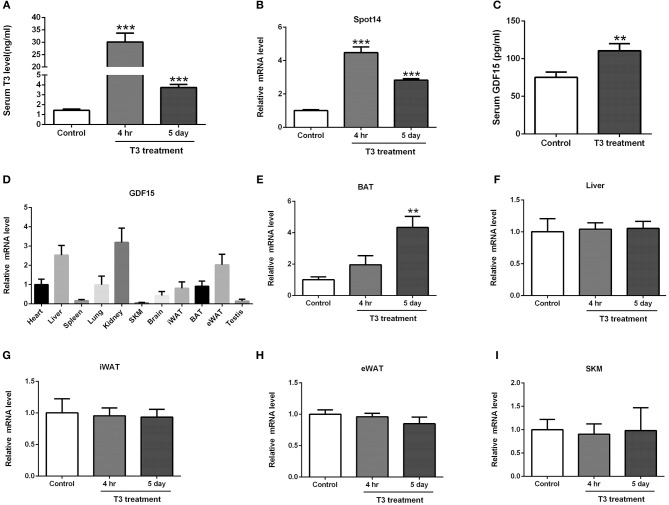
Thyroid hormone induces GDF15 expression in C57BL/6 mice. **(A)** Serum T3 concentrations in mice treated with T3 or vehicle control for 4 h or 5 days. *n* = 5. **(B)** Relative mRNA levels of Spot14 in the liver of mice treated with T3 or vehicle control. *n* = 5. **(C)** Serum GDF15 concentrations in mice treated with T3 or vehicle control for 5 days. **(D)** Quantitative PCR (qPCR) analysis of GDF15 mRNA expression in mouse tissues. *n* = 4. **(E)** Relative mRNA levels of GDF15 in brown adipose tissue (BAT) of mice treated with T3 or vehicle control for 4 hr or 5 days. *n* = 5. **(F–I)** Relative mRNA levels of GDF15 in the liver **(F)**, inguinal white adipose tissue (iWAT) **(G)**, epididymal white adipose tissue (eWAT) **(H)** and skeletal muscle (SKM) **(I)**. *n* = 5. Data are presented as means ± standard deviation (SD). ^**^P < 0.01, ^***^P < 0.001.

Finally, to further determine the effects of thyroid hormone on GDF15, C57BL/6 mice were injected with T3 intraperitoneally for two different time points (4 h or 5 days). As shown in the Figure 2A, serum T3 concentrations were rapidly increased in mice treated with T3 for 4 h and maintained at a high level in mice treated with T3 for 5 days. Besides, it has been well-established that Spot14 could serve as a thyroid hormone responsive gene (23). Consistently, we found that mRNA levels of Spot14 was induced in the liver of mice treated with T3 (Figure 2B). As a result, a 1.47-fold increase of serum GDF15 concentrations was seen at the 5-day time point (*P* < 0.01; Figure 2C). These results suggest that hyperthyroidism was successfully achieved in mice and sufficient to elevate GDF15 in the serum to a similar extent to what was observed in hyperthyroidism patients.

Under physiological conditions, GDF15 mRNA is expressed by a limited number of tissues (22). Our quantitative PCR (qPCR) analysis indicated that GDF15 mRNA expression was higher in liver and kidney, and was also present at a lower level in white adipose tissue (WAT) and brown adipose tissue (BAT; Figure 2D). In contrast, its expression in other tissues, including skeletal muscle, spleen and testis, was relatively low (Figure 2D). We further showed that GDF15 mRNA levels were significantly induced in the BAT of mice treated with T3 (Figure 2E). However, its expression in the liver, inguinal white adipose tissue (iWAT), epididymal white adipose tissue (eWAT), and skeletal muscle (SKM) were not changed (Figures 2F–I).

## Discussion

Previous studies have shown that GDF15 could be up-regulated in response to cellular stress, inflammatory states or environmental factors, to play a protective role in different tissues (22, 24). For instance, circulating GDF15 levels are increased in patients with cancer, cardiovascular disease, muscle atrophy and mitochondrial myopathy (24–27). Besides, vigorous submaximal exercise increases circulating GDF15 levels in humans and long-term fasting up-regulates its gene expression via activating IRE1α-XBP1s signaling in the livers of mice (28, 29). Moreover, serum GDF15 levels are increased in obese and type 2 diabetic patients and correlated with BMI, body fat, plasma glucose and C-reactive proteins (30, 31). In addition, GDF15 levels are identified as a biomarker for the use of metformin in type 2 diabetes and its concentration reflects the dose of metformin (32). However, physiological and pathological factors that modulate GDF15 expression are still obscure.

In the present study, we uncovered for the first time to our knowledge that thyroid hormone plays an important role in the regulation of GDF15 expression. This is supported by several lines of evidence. Firstly, we found that circulating GDF15 levels were markedly elevated in patients with hyperthyroidism compared with healthy subjects and dramatically declined after thionamide treatment. Secondly, logistic regression analysis confirmed an independent association between serum GDF15 levels and hyperthyroidism. Besides, serum GDF15 levels were inversely correlated with total cholesterol levels, which is also observed in 147 older people with hypertension and in 2,991 participants in the Framingham Offspring Study who were free of clinically overt cardiovascular disease (33, 34). Thirdly, we showed that expression levels of GDF15 were induced in mice by T3 treatment. Therefore, our results provide the clinical and animal evidence that GDF15 is associated with hyperthyroidism. However, the sample size was relatively small and data from larger clinical patients are needed.

Further studies are still needed to clarify the relationship between thyroid hormone and GDF15. First, one of the key roles of thyroid hormone is to up-regulate UCP1 expression in BAT to increase adaptive thermogenesis and counteract obesity, which overlaps with the action of GDF15. Therefore, future studies should determine whether GDF15 is necessary for some of the thyroid hormone-associated metabolic benefits, such as increasing energy expenditure. It would also be interesting to explore whether the neutralizing antibody against GDF15 could attenuate the hyperthyroidism-associated emaciation. Second, GDF15 was shown to suppress food intake, whereas hyperthyroidism is known to increase appetite. It is tempting to speculate that the orexigenic nature of thyroid hormone could be independent of GDF15. On the other hand, some studies reported that anorexia is observed in the older hyperthyroid patients (35, 36), suggesting the necessity of measurement of GDF15 in this age group. Third, the reason for the tissue-specific effects of T3 on GDF15 expression in mice remains unknown. Our animal results showed that BAT might be a source for increased circulating GDF15 concentrations upon T3 treatment. Recently, substantial depots of functionally active brown adipose tissue were identified in adult humans (37–39), and are increased or activated by several pathophysiological states, including hyperthyroidism (40). Given T3 is a strong stimulator in converting WAT to “beige” (41), a longer term of T3 treatment might induce GDF15 expression in WAT as well. Besides, we cannot rule out the possibility that other tissues not studied here might also contribute to the increased serum GDF15 during hyperthyroidism. Fourth, whether thyroid hormone regulates the secretion of GDF15 needs to be determined. Previous studies have shown that unprocessed GDF15 precursor is rapidly secreted, while mature GDF15 generated within the cell by intracellular processing is secreted much slower (22, 42). Usually, only the mature form of GDF15 is secreted, but under some pathological conditions, the full-length form is also secreted (22). Finally, considering that GDF15 may be a more general cell stress-response cytokine (22, 24), we speculate that in addition to thyroid hormone, other hormones and nutrients, could regulate GDF15 expression under different physiological and stress conditions. Moreover, whether circulating GDF15 concentrations were altered in other thyroid disorders, such as hypothyroid, toxic thyroid adenoma, pituitary TSH adenoma, and resistance to thyroid hormone (RTH), remains unknown.

In summary, our study demonstrated that thyroid hormone could increase GDF15 expression and concentrations in mice and humans. To our knowledge, these observations are being made for the first time that GDF15 is independently associated with hyperthyroidism.

## Author Contributions

XX and JJ conceived the project. JZ and ML performed the experiments. YC, SZ, and ZS took statistical analysis. YL and JJ drafted the manuscript. HY, XL, XX, and JJ handled funding and supervision. All authors reviewed the manuscript. XX and JJ are the guarantors of this work and, as such, had full access to all the data in the study and take responsibility for the integrity of the data and the accuracy of the data analysis.

### Conflict of Interest Statement

The authors declare that the research was conducted in the absence of any commercial or financial relationships that could be construed as a potential conflict of interest.

## Abbreviations

BAT: Brown adipose tissue
DBP: Diastolic blood pressure
ELISA: Enzyme-linked immunosorbent assay
GDF15: Growth differentiation factor 15
HDL: High-density lipoprotein
SBP: Systolic blood pressure
SD: Standard deviation
SKM: Skeletal muscle
TBIL: Total bilirubin
TC: Total cholesterol
WAT: White adipose tissue.

## References

[1] Sinha RA Singh BK Yen PM. Direct effects of thyroid hormones on hepatic lipid metabolism. Nat Rev Endocrinol. (2018) 14:259–69. 10.1038/nrendo.2018.10 29472712 PMC6013028

[2] Cheng SY Leonard JL Davis PJ. Molecular aspects of thyroid hormone actions Endocr Rev. (2010) 31:139–70. 10.1210/er.2009-0007 20051527 PMC2852208

[3] Ribeiro MO. Effects of thyroid hormone analogs on lipid metabolism and thermogenesis. Thyroid (2008) 18:197–203. 10.1089/thy.2007.0288 18279020

[4] Lee JY Takahashi N Yasubuchi M Kim YI Hashizaki H Kim MJ. Triiodothyronine induces UCP-1 expression and mitochondrial biogenesis in human adipocytes. Am J Physiol Cell Physiol. (2012) 302:C463–72. 10.1152/ajpcell.00010.2011 22075692

[5] Jansen MS Cook GA Song S Park EA Thyroid hormone regulates carnitine palmitoyltransferase I alpha gene expression through elements in the promoter and first intron. J Biol Chem. (2000) 275:34989–97. 10.1074/jbc.M001752200 10956641

[6] Xiao F Lin M Huang P Zeng J Zeng X Zhang H. Elevated serum fibroblast growth factor 21 levels in patients with hyperthyroidism. J Clin Endocrinol Metab. (2015) 100:3800–5. 10.1210/jc.2015-1797 26241324

[7] Adams AC Astapova I Fisher FM Badman MK Kurgansky KE Flier JS. Thyroid hormone regulates hepatic expression of fibroblast growth factor 21 in a PPARalpha-dependent manner. J Biol Chem. (2010) 285:14078–82. 10.1074/jbc.C110.107375 20236931 PMC2863226

[8] Bakiner O Bozkirli E Ertugrul D Sezgin N Ertorer E. Plasma fetuin-A levels are reduced in patients with hypothyroidism. Eur J Endocrinol. (2014) 170:411–8. 10.1530/EJE-13-0831 24366942

[9] Ruchala M Zybek A Szczepanek-Parulska E. Serum irisin levels and thyroid function–newly discovered association. Peptides (2014) 60:51–5. 10.1016/j.peptides.2014.07.021 25102448

[10] Bootcov MR Bauskin AR Valenzuela SM Moore AG Bansal M He XY. MIC-1, a novel macrophage inhibitory cytokine, is a divergent member of the TGF-beta superfamily. Proc Natl Acad Sci USA. (1997) 94:11514–9. 9326641 10.1073/pnas.94.21.11514 PMC23523

[11] Tsai VW Lin S Brown DA Salis A Breit SN Anorexia-cachexia and obesity treatment may be two sides of the same coin: role of the TGF-β superfamily cytokine MIC-1/GDF15. Int J Obes. (2016) 40:193–7. 10.1038/ijo.2015.242 26620888

[12] Johnen H Lin S Kuffner T Brown DA Tsai VW Bauskin AR. Tumor-induced anorexia and weight loss are mediated by the TGF-beta superfamily cytokine MIC-1. Nat Med. (2007) 13:1333–40. 10.1038/nm1677 17982462

[13] Mullican SE Lin-Schmidt X Chin CN Chavez JA Furman JL Armstrong AA. GFRAL is the receptor for GDF15 and the ligand promotes weight loss in mice and nonhuman primates. Nat Med. (2017) 23:1150–7. 10.1038/nm.4392 28846097

[14] Emmerson PJ Wang F Du Y Liu Q Pickard RT Gonciarz MD. The metabolic effects of GDF15 are mediated by the orphan receptor GFRAL. Nat Med. (2017) 23:1215–9. 10.1038/nm.4393 28846098

[15] Yang L Chang CC Sun Z Madsen D Zhu H Padkjær SB. GFRAL is the receptor for GDF15 and is required for the anti-obesity effects of the ligand. Nat Med. (2017) 23:1158–66. 10.1038/nm.4394 28846099

[16] Hsu JY Crawley S Chen M Ayupova DA Lindhout DA Higbee J. Non-homeostatic body weight regulation through a brainstem-restricted receptor for GDF15. Nature (2017) 550:255–9. 10.1038/nature24042 28953886

[17] Chrysovergis K Wang X Kosak J Lee SH Kim JS Foley JF. NAG-1/GDF-15 prevents obesity by increasing thermogenesis, lipolysis and oxidative metabolism. Int J Obes. (2014) 38:1555–64. 10.1038/ijo.2014.27 24531647 PMC4135041

[18] Tsai VW Zhang HP Manandhar R Lee-Ng KKM Lebhar H Marquis CP. Treatment with the TGF-b superfamily cytokine MIC-1/GDF15 reduces the adiposity and corrects the metabolic dysfunction of mice with diet-induced obesity. Int J Obes. (2018) 42:561–71. 10.1038/ijo.2017.258 29026214

[19] Xiong Y Walker K Min X Hale C Tran T Komorowski R. Long-acting MIC-1/GDF15 molecules to treat obesity: evidence from mice to monkeys. Sci Transl Med. (2017) 9:412. 10.1126/scitranslmed.aan8732 29046435

[20] Tsai VW Macia L Johnen H Kuffner T Manadhar R Jørgensen SB. TGF-b superfamily cytokine MIC-1/GDF15 is a physiological appetite and body weight regulator. PLoS ONE (2013) 8:e55174. 10.1371/journal.pone.0055174 23468844 PMC3585300

[21] Tran T Yang J Gardner J Xiong Y. GDF15 deficiency promotes high fat diet-induced obesity in mice. PLoS ONE (2018) 13:e0201584. 10.1371/journal.pone.0201584 30070999 PMC6072047

[22] Tsai VWW Husaini Y Sainsbury A Brown DA Breit SN. The MIC-1/GDF15-GFRAL pathway in energy homeostasis: implications for obesity, cachexia, and other associated diseases. Cell Metab. (2018) 28:353–68. 10.1016/j.cmet.2018.07.018 30184485

[23] Rudolph MC Wellberg EA Lewis AS Terrell KL Merz AL Maluf NK. Thyroid hormone responsive protein Spot14 enhances catalysis of fatty acid synthase in lactating mammary epithelium. J Lipid Res. (2014) 55:1052–65. 10.1194/jlr.M044487 24771867 PMC4031937

[24] Adela R Banerjee SK. GDF-15 as a target and biomarker for diabetes and cardiovascular diseases: a translational prospective. J Diabetes Res. (2015) 2015:490842. 10.1155/2015/490842 26273671 PMC4530250

[25] Yatsuga S Fujita Y Ishii A Fukumoto Y Arahata H Kakuma T. Growth differentiation factor 15 as a useful biomarker for mitochondrial disorders. Ann Neurol. (2015) 78:814–23. 10.1002/ana.24506 26463265 PMC5057301

[26] Ji X Zhao L Ji K Zhao Y Li W Zhang R. Growth differentiation factor 15 is a novel diagnostic biomarker of mitochondrial diseases. Mol Neurobiol. (2017) 54:8110–6. 10.1007/s12035-016-0283-7 27889897

[27] Wollert KC Kempf T Wallentin L. Growth differentiation factor 15 as a biomarker in cardiovascular disease. Clin Chem. (2017) 63:140–51. 10.1373/clinchem.2016.255174 28062617

[28] Kleinert M Clemmensen C Sjøberg KA Carl CS Jeppesen JF Wojtaszewski JFP. Exercise increases circulating GDF15 in humans. Mol Metab. (2018) 9:187–91. 10.1016/j.molmet.2017.12.016 29398617 PMC5870087

[29] Zhang M Sun W Qian J Tang Y. Fasting exacerbates hepatic growth differentiation factor 15 to promote fatty acid β-oxidation and ketogenesis via activating XBP1 signaling in liver. Redox Biol. (2018) 16:87–96. 10.1016/j.redox.2018.01.013 29482168 PMC5952356

[30] Dostálová I Roubícek T Bártlová M Mráz M Lacinová Z Haluzíková D. Increased serum concentrations of macrophage inhibitory cytokine-1 in patients with obesity and type 2 diabetes mellitus: the influence of very low calorie diet. Eur J Endocrinol. (2009) 161:397–404. 10.1530/EJE-09-0417 19515791

[31] Vila G Riedl M Anderwald C Resl M Handisurya A Clodi M. The relationship between insulin resistance and the cardiovascular biomarker growth differentiation factor-15 in obese patients. Clin Chem. (2011) 57:309–16. 10.1373/clinchem.2010.153726 21164037

[32] Gerstein HC Pare G Hess S Ford RJ Sjaarda J Raman K. Growth differentiation factor 15 as a novel biomarker for metformin. Diabetes Care (2017) 40:280–3. 10.2337/dc16-1682 27974345

[33] Ho JE Mahajan A Chen MH Larson MG McCabe EL Ghorbani A. Clinical and genetic correlates of growth differentiation factor 15 in the community. Clin Chem. (2012) 58:1582–91. 10.1373/clinchem.2012.190322 22997280 PMC4150608

[34] Barma M Khan F Price RJG Donnan PT Messow CM Ford I. Association between GDF-15 levels and changes in vascular and physical function in older patients with hypertension. Aging Clin Exp Res. (2017) 29:1055–9. 10.1007/s40520-016-0636-0 27734214 PMC5589783

[35] Trivalle C Doucet J Chassagne P Landrin I Kadri N Menard JF. Differences in the signs and symptoms of hyperthyroidism in older and younger patients. J Am Geriatr Soc. (1996) 44:50–3. 8537590 10.1111/j.1532-5415.1996.tb05637.x

[36] Dai WX Meng XW. Causes of anorexia in untreated hyperthyroidism: a prospective study. Postgrad Med J. (2000) 76:292–4. 10.1136/pmj.76.895.292 10775283 PMC1741582

[37] van Marken Lichtenbelt WD Vanhommerig JW Smulders NM Drossaerts JM Kemerink GJ Bouvy ND. Cold-activated brown adipose tissue in healthy men. N Engl J Med. (2009) 360:1500–8. 10.1056/NEJMoa0808718 19357405

[38] Cypess AM Lehman S Williams G Tal I Rodman D Goldfine AB. Identification and importance of brown adipose tissue in adult humans. N Engl J Med. (2009) 360:1509–17. 10.1056/NEJMoa0810780 19357406 PMC2859951

[39] Virtanen KA Lidell ME Orava J Heglind M Westergren R Niemi T. Functional brown adipose tissue in healthy adults. N Engl J Med. (2009) 360:1518–25. 10.1056/NEJMoa0808949 19357407

[40] Lahesmaa M Orava J Schalin-Jäntti C Soinio M Hannukainen JC Noponen T. Hyperthyroidism increases brown fat metabolism in humans. J Clin Endocrinol Metab. (2014) 99:E28–35. 10.1210/jc.2013-2312 24152690

[41] Obregon MJ. Adipose tissues and thyroid hormones. Front Physiol. (2014) 5:479. 10.3389/fphys.2014.00479 25566082 PMC4263094

[42] Bauskin AR Jiang L Luo XW Wu L Brown DA Breit SN. The TGF-beta superfamily cytokine MIC-1/GDF15: secretory mechanisms facilitate creation of latent stromal stores. J Interferon Cytokine Res. (2010) 30:389–97. 10.1089/jir.2009.0052 20187768

